# How Tourist Preference and Satisfaction Can Contribute to Improved Welfare Standards at Elephant Tourism Venues in Thailand

**DOI:** 10.3390/ani11041094

**Published:** 2021-04-12

**Authors:** Emily K. Flower, Georgette Leah Burns, Darryl N. Jones

**Affiliations:** Environmental Futures Research Institute and School of Environment and Science, Griffith University, Brisbane 4111, Australia; leah.burns@griffith.edu.au (G.L.B.); d.jones@griffith.edu.au (D.N.J.)

**Keywords:** tourist satisfaction, tourist preference, animal welfare, Asian elephant

## Abstract

**Simple Summary:**

Tourists who visit elephant tourism venues (ETVs), and other wildlife attractions, can influence the activities offered at these venues by positive and negative word-of-mouth. When a person has a good experience and they tell their family and friends, or leave reviews online, this can influence people to participate in the activity. The same is also true when a person has a bad experience; although, in this instance, they can influence people not to participate in the activity, limiting the possible success of that venue. Generally, tourists seem to prefer to visit ETVs that allow elephants to behave naturally over those that make their elephants entertain tourists. Therefore, we visited twelve ETVs in Chiang Mai, Thailand, and compared the opinions of tourists who visited riding and non-riding venues towards the use of captive elephants for entertainment. We found that tourists who reported a preference for ETVs with higher welfare standards had personally seen elephants being treated poorly. Additionally, higher welfare standards may positively affect tourists’ satisfaction with their experience. Therefore, tourists can influence the activities offered at ETVs by demanding better living conditions for elephants and only financially supporting ETVs with higher welfare standards.

**Abstract:**

Consumer satisfaction and preference can be integral in influencing and solidifying change in user-driven industries such as tourism. High satisfaction rates are imperative to the continual success of a venue as satisfaction determines the likelihood of repeat business and positive recommendations to friends, family and online review forums. Tourist preference for ecocentric tourism venues, over anthropocentric ones, appears to be increasing in elephant tourism venues (ETVs) in Thailand. To explore this, we visited twelve ETVs in Chiang Mai, Thailand, and compared the preferences and satisfaction of tourists who visited riding and non-riding venues toward the use of captive elephants in an entertainment setting. We found that tourists visited riding and non-riding ETVs for similar reasons, primarily due to recommendations from friends and reviews, and because the venue had a good reputation. Tourist preference for higher welfare standards was observed at venues where participants directly observed poor treatment of the elephants. Tourist satisfaction may be impacted by higher elephant welfare standards; therefore, tourists have the ability to influence the elephant tourism industry by demanding better living conditions for elephants and only financially supporting ETVs with higher welfare standards.

## 1. Introduction

In 2018, global wildlife tourism generated over five times the income of the illegal wildlife trade [1]. This highlights the economic importance of the wildlife tourism industry and is a significant argument for protecting wildlife. In the same year, Thailand’s travel and tourism was one of the country’s highest-earning sectors, attracting 38 million international tourists [2] and contributing 21.6% to the nation’s gross domestic product (GDP) [3]. Increasing in parallel with the rise of international visitors to Thailand is the rise in demand for animal-based tourism, particularly for large, charismatic species such as the elephant. Simultaneously, tourists’ awareness of the potential poor treatment of animals used in these activities, such as the misuse or overuse of a bull hook and the unnatural activities performed by elephants, is also on the rise [4,5].

In recent years, the poor treatment and welfare of animals used in wildlife tourism attractions has resulted in considerable outrage and motivated calls for action (for example, [6]). Media coverage and public concern expressed in social media against animal mistreatment has grown [7]. The treatment of animals within tourism and the nature of the interactions between animals and people within these venues can be influenced by a person’s predominant worldview. Two common environmental worldviews, anthropocentrism and ecocentrism, are structured around whether or not humans are considered to occupy the centre of moral concern. An anthropocentric, or human-centred, worldview holds that nature and wildlife are commodified and are valued primarily by their use to humans. This worldview is predominant in Western societies and assumes that humans are the preeminent species, superior to all others. Conversely, an ecocentric, or life-centred, worldview holds that all life has intrinsic value, regardless of its relationship to humans. This worldview accepts that humans are not dominant over, nor separate from, other species and acknowledges that nature exists irrespective of humanity [8]. People’s attitudes towards wildlife vary due to many factors, such as their level of education and cultural differences; however, the higher the value people assign to a particular species, the more likely they are to be motivated to protect it [9].

Fennell [10] (p. 326) deems the link between tourism and animal ethics as essential, suggesting that animal welfare, as one aspect of animal ethics, is of concern for venues as “animals that are fit and healthy are, quite simply, better for business”. Fennell, however, further argues that the moral acceptability regarding the use of, and level of care afforded to animals within tourism should be questioned. In the context of elephant tourism in Thailand, many elephants are kept in inadequate conditions, which prevent them from engaging in normal species-specific behaviours and cannot fulfil their basic needs [11,12]. Conditions common in elephant tourism venues (ETVs), such as painful training methods, no variation in diet, insufficient socialisation and stereotypic behaviour, can have negative physical and psychological effects on individual elephants [11,13]. These conditions are currently widespread in ETVs and make higher welfare standards for all captive elephants in Thailand not possible as most venues are unable to maintain a setting that adequately replicates an elephant’s natural environment [11,12,14]. Importantly, the success of an ETV and the welfare of its elephants are linked. An individual elephant’s welfare is dependent upon the financial success of the ETV, which is in turn dependent on the elephant’s ability to participate in interactions and thus attract visitors to the venue [14].

Severe training methods are employed to enable elephants to be ridden; thus, the presence of riding at an ETV can be indicative of the long-term treatment of the elephants [11]. Although riding is only one aspect of captive elephant welfare, it is an important aspect. Animal welfare organisations publicising welfare concerns associated with elephant riding (for example, [15]), have increased public awareness and potentially contributed to a decline in the number of tourists visiting riding venues. However, many tourists continue to show preference for trek types that enable them to ride elephants. A 2016 study found that 40% of tourists surveyed in Thailand had ridden, or planned to ride, an elephant during their visit, equating to approximately 12.8 million elephant rides [15]. For these reasons, this research focuses on preferences for trek types in ETVs as a measure of tourist satisfaction with welfare standards.

Tourist satisfaction occurs when the actual experience matches or surpasses the expectations for the experience, resulting in an individual’s pleasurable feeling of fulfilment [16,17]. Tourist preference indicates visitors’ perceptions and judgements of an experience after an actual visitation, and can be positive, negative or neutral [18]. Satisfaction and preference influence and solidify change in consumer-driven industries, such as tourism, as the success of a venue is highly dependent on tourists’ satisfaction with its activities and on their willingness to pay for these activities [19]. High satisfaction rates are imperative to the continual success of a venue as satisfaction determines the likelihood of repeat business and positive word-of-mouth recommendations to friends, family and online review forums [20,21]. Tourist satisfaction may be measured using multiple methods; for example, the push-pull satisfaction approach which asks the tourist to report their motivations for the visit (push factors) and to rate the degree with which they were satisfied with the venue’s activities (pull factors) [22].

While tourists have the ability to influence the elephant tourism industry by providing feedback regarding their attitudes towards the treatment of elephants at ETVs, a gap exists for some between their attitude or values and their actual behaviour [23]. This process, known as cognitive dissonance, is particularly prominent in elephant tourism, where tourists appear to disengage from their moral and ethical values, disguise the moral implications of their decision, and consequently permit themselves to engage in an experience that they would usually consider ‘wrong’ [10,24]. This could occur because they find the interaction (for example elephant riding) extremely attractive, or because tourists are less likely to consider the implications of their choices while on holiday [25].

While considerable research has been undertaken on animal welfare in tourism [10,26], the influence of welfare concerns among tourists participating in wildlife-based tourism remains poorly understood [27]. This is particularly pertinent for studies concerning tourists’ ethical standpoints for the Asian elephant [5]. Further, research on the welfare implications of elephants living in captivity, including investigation of the impacts of elephant riding (for example, [28,29,30,31]) has recently increased. However, the exploration of tourist attitudes towards elephant riding, an important factor for the popularity and longevity of riding as an activity offered at ETVs, is insufficient [32].

This study compares the preferences and satisfaction of tourists who visited elephant riding and non-riding venues in Thailand. Specifically, we identify and analyse themes from visitor responses concerning their most preferred and least preferred activity during their time at an ETV, their intention to ride an elephant in future and re-visit the ETV and their reason for believing the ETV they visited does or does not treat its elephants appropriately. We also discuss how increased tourist demand for ETVs which are more ecocentric, in that they do not offer riding or activities such as elephant shows, may lead to higher welfare standards in the elephant tourism industry.

## 2. Materials and Methods

### 2.1. Study Area

**Figure 1:**
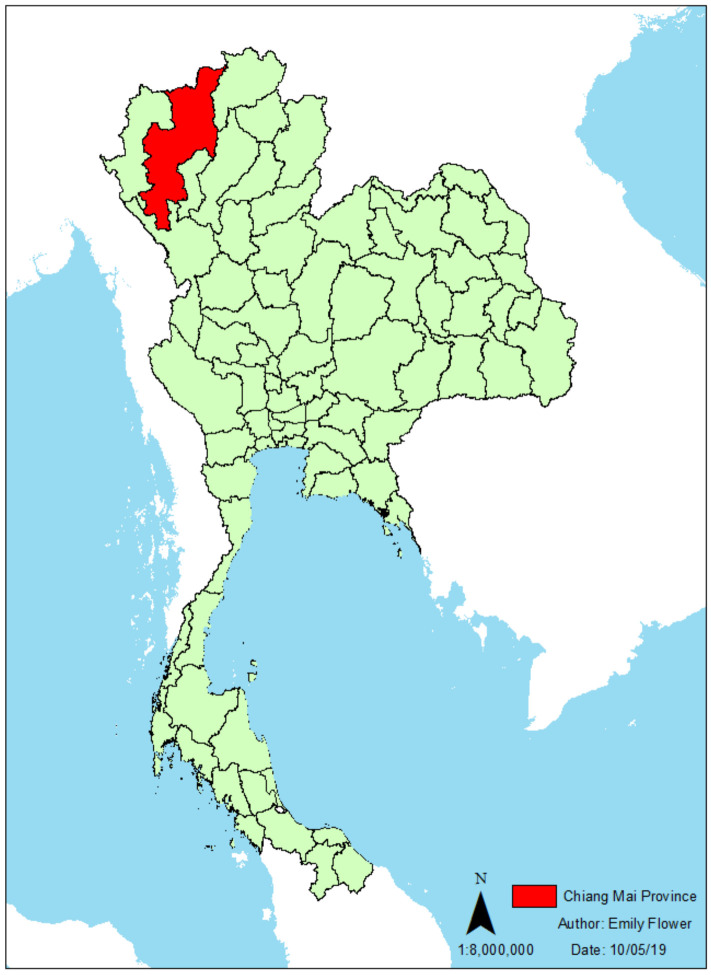
Location of Chiang Mai Province (red area) within Thailand.

Field research for this study occurred in Chiang Mai, Thailand (Figure 1). Experiencing elephant tourism activities is a major motivation for tourists visiting Thailand, particularly in Chiang Mai Province which holds the highest number of ETVs nationwide [33]. In 2017, the Thai National Institute of Elephant Research and Health Services reported that approximately 2700 elephants were living in 223 tourism venues throughout Thailand, with an additional 1000 being used for logging and farming activities [34].

### 2.2. Sampling

Data were collected at twelve ETVs in January 2018 during a period with typically high international visitation [2]. Venues were selected based on the information advertised on their websites, with the intention to sample across ETVs offering a range of activities. For this study, venues were separated into three groups dependent on the trek type offered: non-riding, bareback riding and seated riding. Although we attempted to visit equal proportions of ETVs with all three trek types, more tourists were surveyed at non-riding venues compared to seated riding venues. This skewed sampling was due to multiple factors. Prior to data collection, to ensure transparency, the researcher disclosed the intent to observe tourist-elephant interactions and requested permission from ETV staff. Most seated riding venues either did not respond or declined the opportunity to take part. During the time between booking and the actual visit, one ETV had removed seated riding from the activities they offer. On the day we collected data at the seated riding venue, most visitors were non-English speaking and were unwilling or unable to participate. In addition, at the seated riding venue the researcher was unable to interact with other tourists. This all led to a decrease in the tourists sampled at the seated riding venues. Further, non-riding venues could allocate more than one or two tourists per elephant, enabling a greater number of potential participants at those venues.

Participants were surveyed at non-riding venues (*n* = 106), bareback riding venues (*n* = 23) and a seated riding venue (*n* = 3) (see Supplementary Materials Table S5 for venue participant numbers). All 132 participants were international visitors to Thailand. Participants were recruited using convenience sampling to maximise the possible participant pool. Inclusion was voluntary as potential participants were approached during transit to the ETV and asked if they would be willing to participate. Thai tourists were not surveyed during this study because they were not observed at an ETV or they did not require transportation assistance to visit an ETV. To enable pairing of their pre- and post-visit questionnaires for analysis, participants were instructed to include their initials and year of birth on both surveys. The name of each ETV was also documented on questionnaires prior to distribution to allow comparisons between venues. Over the sampling period, 132 completed paired questionnaires were returned.

### 2.3. Questionnaire Distribution and Design

Most sampling sessions were arranged directly with the ETVs via email (see Supplementary Materials—ETV Recruitment Email Script) before visiting Thailand, although some visits were booked whilst in Thailand via email and phone. The researcher practised participant observation by undertaking all activities with the tourists, including transportation to and from the venue, and participating in two (when possible) full-day visits to each ETV. Some ETVs were not visited twice because of misunderstandings of transportation or because, for those booked in Thailand, there was insufficient time for a second visit during the available fieldwork period.

Information on tourist preference and satisfaction regarding elephant-related activities was obtained through questionnaires. Two English language questionnaires, one pre-visit (32 questions) and one post-visit (29 questions), were offered to the same participants by the researcher and completed during transit to and from the ETV (see Supplementary Materials—Questionnaires and Supplementary Materials—Tourist Questionnaire Information Sheet). The questionnaire’s design largely followed that developed by Rattan, Eagles and Mair [35]. The pre-visit questionnaire contained questions regarding participant demographics and tourist motivation, including participants’ reasons for visiting the ETV, the elephant-related activities they wished to experience, and whether they had visited an ETV previously. The post-visit questionnaire contained questions focused on tourist satisfaction, including the participants’ most preferred and least preferred activities, their reasons for choosing those activities, their reason for whether they would ride an elephant in the future, whether they believed the elephants were treated appropriately, and if they would visit the ETV again. These were a combination of both open-ended and multiple-choice questions.

### 2.4. Data Analyses

Quantitative data (for example, multiple-choice questions) were entered into Microsoft Excel and qualitative data (for example, open-ended questions) were imported into NVivo 12 Pro [36]. Prior to analysis, data were screened per question and duplicate responses removed. Specifically, when participants ticked one of the multiple-choice answers and then provided this same response in the ’other’ short response section the multiple-choice selection was removed, and the ’other’ short response retained. Simple frequencies were performed on the quantitative data in Microsoft Excel to determine the number of participants who responded to each question and how they responded. The open-ended responses were imported into NVivo and themes were constructed per question based on respondents’ answers using thematic analysis (see Supplementary Materials for all themes). Thematic analysis identifies, analyses and reports patterns within data [37]. Frequencies for open-ended questions, where participants could provide more than one response, were calculated using the number of responses received, not the number of tourists who participated. Hence, the term ’respondents’ refers to a subset of the study sample and is used rather than ’participants’ when discussing each question’s responses in the results.

## 3. Results

### 3.1. Reason for Visiting the ETV

When coded for similarities, 129 respondents gave 11 reasons for visiting an ETV (Table 1). Six choices were provided with a ‘tick all that apply’ format, in addition to an ‘other’ short response section. Five additional themes were constructed from respondents’ short response answers. Two respondents wanted to ride an elephant bareback at an ETV that “is safe for them” and “takes care of animals”. The most popular reason stated for visiting the ETV was recommendations from friends or reviews (*n* = 60, 26.2%), closely followed by the good reputation of the ETV (*n* = 59, 25.8%). Additional popular motivational factors for respondents included the decision that the ETV best suited their needs after completing research on elephant parks in the area (*n* = 44, 19%) and because it was advertised by a local travel agent (*n* = 24, 10.5%).

**Figure 2:**
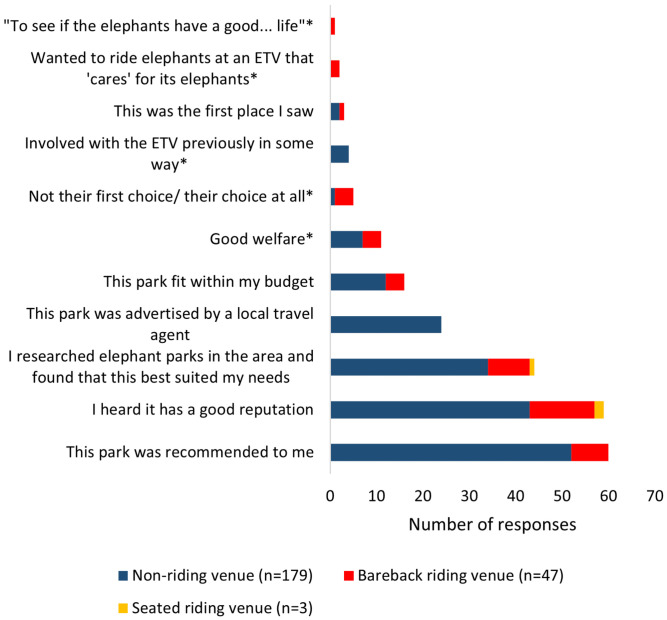
Respondent reasons, per venue type, for choosing the ETV they visited. ’*’ Denotes themes constructed from participants’ responses, all others were reasons included in the questionnaire.

When inspecting these reasons per venue type (Figure 2), respondents who visited a non-riding venue most commonly said they visited the ETV because it was recommended to them (*n* = 52, 29%) or because they heard it had a good reputation (*n* = 43, 24%). Respondents who visited a bareback riding venue said they visited the ETV because they heard it had a good reputation (*n* = 14, 30%) or because they researched elephant parks in the area and found that the particular ETV best suited their needs (*n* = 9, 19%). Two-thirds of these respondents who visited a venue with seated rides said they heard it had a good reputation (*n* = 2).

**Figure 3:**
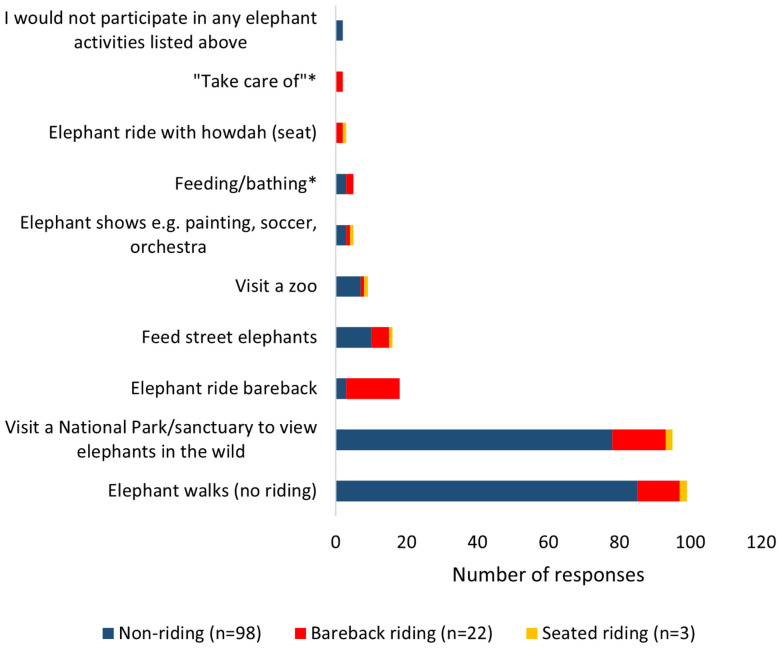
Respondent preferences for elephant-related activities per venue type. ’*’ Denotes themes constructed from participants’ responses, all others were reasons included in the questionnaire. Total of 254 activities were stated (participants could give >1 activity).

When coded for similarities, 123 respondents provided ten elephant-related activities they either had participated in or wanted to participate in while in Thailand (Figure 3). Eight choices were provided with a ‘tick all that apply’ format, in addition to an ‘other’ short response section. Two additional themes were constructed from respondents’ short response answers. Wanting to walk with an elephant was the most popular activity (*n* = 99, 39%) followed by wanting to visit a national park or sanctuary to view elephants (*n* = 95, 37%).

When examining preferred activities based on venue type, respondents from non-riding venues preferred participating in an elephant walk (*n* = 85, 44.5%) or visiting a national park or sanctuary to view elephants in the wild (*n* = 78, 40.8%). More respondents who visited bareback riding venues preferred to ride an elephant bareback or visit a national park or sanctuary to view elephants in the wild (both *n* = 15, 27.3%) than to walk with an elephant (*n* = 12, 21.8%). One quarter of responses from those participants who visited an ETV with seated rides wanted to participate in an elephant walk and wanted to visit a national park or sanctuary to view elephants in the wild (both *n* = 2). Only one respondent from this venue reportedly wanted to ride an elephant with a *howdah* (a traditional seat used to ride an elephant).

### 3.2. Most Preferred Activities

**Figure 4:**
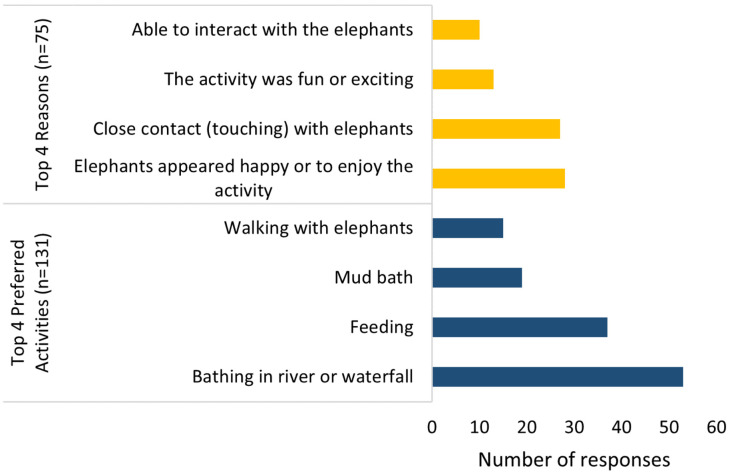
Respondents’ top four preferred activities (**bottom**) and the top four reasons respondents gave for choosing their preferred activity (**top**). 169 total activities and 123 total reasons stated (participants could give >1 activity and reason).

Although 131 people stated their preferred activity, only 75 provided a reason for why they enjoyed the activity (Figure 4). As this was an open-ended question, some respondents provided more than one preferred activity and more than one reason for liking an activity, resulting in 169 preferred activities and 123 reasons for liking the activities (21 themes when coded, see Supplementary Materials Table S1).

Six respondents (five from a bareback riding venue and one from a seated riding venue) reported riding as one of their preferred activities because “The feeling was great to ride an elephant. Like a King” and it was a “unique” and “up close” experience. Bathing elephants in water was reported as the overall preferred activity (*n* = 53, 31.4%) because respondents perceived that the elephants enjoyed this activity (*n* = 10) (“The animals seemed to enjoy that the most”), they were able to be in close contact with the elephants (*n* = 9), and the activity was considered fun (*n* = 7). Feeding the elephants was the second most preferred activity (*n* = 37, 21.9%) due to close contact with the elephants (*n* = 9) (“opportunity to get close and meet each of the elephants and stroke them”), belief that the elephants enjoyed this activity (*n* = 6), and that the elephants were able to behave naturally (*n* = 3) (“in their most natural state”).

The reasons respondents stated for preferred activities, even when the activities differed, were often similar (Figure 4). The most common reason for liking any activity was because the elephants appeared happy or to enjoy the activity (*n* = 28, 22.8%) (“I liked (the activities) best because I could be sure the elephants were enjoying themselves as well”), followed by the ability for close contact with the elephants (*n* = 27, 21.95%) (“I loved getting very personal with them”). Subsequent reasons for liking an activity were because the activity was fun or exciting (*n* = 13, 10.6%) or due to the ability to interact with the elephants (*n* = 10, 8.1%).

### 3.3. Least Preferred Activities

**Figure 5:**
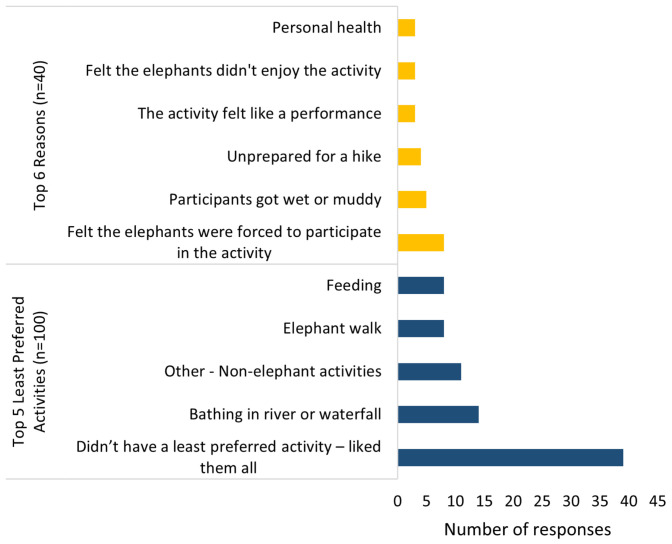
Respondents’ top five least preferred activities (**bottom**) and the top six reasons respondents gave for choosing their least preferred activity (**top**). 147 total activities and 49 total reasons stated (participants could give >1 activity and reason).

Although 100 people stated their least preferred activity, only 40 people provided a reason for why they disliked the activity (Figure 5). As this was an open-ended question, some respondents provided more than one least preferred activity and more than one reason for disliking an activity, resulting in 147 least preferred activities and 49 reasons for disliking the activities (19 themes when coded, see Supplementary Materials Table S2).

Only eight respondents (seven from a bareback riding venue and one from a seated riding venue) reported riding as their least preferred activity because the elephants are “yelled at constantly” and “they aren’t in (their) natural habitat”. The most common response was that respondents liked all activities and did not have a least preferred one (*n* = 39, 26.5%). Bathing elephants in water was reported as the least popular actual activity (*n* = 14, 9.5%) because the elephants were forced to do the activity (*n* = 5) (“…unsure how much the elephants were forced to do it…I fear this is just another form of performance”), respondents got wet or muddy (*n* = 3), and they thought the elephants did not enjoy the activity (*n* = 2). The next most disliked activities were non-elephant related (*n* = 11, 7.5%), such as the long journey to the ETV (*n* = 2), a fear of sickness (*n* = 1) and one respondent would have liked to spend more time walking instead of having a long lunch. Walking with elephants and feeding were the subsequent least preferred activities (both *n* = 8, 5.4%) because the respondents were unprepared for a hike or the elephants were chained for prolonged periods of time (“…they were tied up and it felt like some tourist park”).

Again, the respondents had common reasons for disliking different activities (Figure 5). No further reasons were provided by those respondents who did not have a least preferred activity; however, of those respondents who did provide reasons, the most common was because they felt the elephants were forced to do the activity (*n* = 8, 16.3%), they disliked getting wet or muddy during the activity (*n* = 5, 10.2%), and they were unprepared for a hike (*n* = 4, 8.2%). Additional reasons for disliking an activity were due to the activity feeling like a performance, respondent’s perceptions that the elephants did not enjoy the activity, and for personal health (all *n* = 3, 6.1%).

### 3.4. Preferences for Elephant Riding

Due to the reasons outlined in the methods, 80% (*n* = 106) of participants were surveyed at non-riding venues; however, four participants who visited a venue with bareback riding chose not to participate in that activity (totalling *n* = 110). Nineteen participants participated in bareback riding, and three participated in seated riding.

From 113 respondents, 83% said they would not ride an elephant in future (*n* = 94), and 17% said that they would (*n* = 19; see Supplementary Materials Table S3). Of the 19 respondents who would ride an elephant in future, only six stipulated the ride should be bareback. All others did not distinguish between a bareback or seated ride. Only one of the three participants who participated in a seated ride said they would ride an elephant again (without stipulating bareback or seated). Four respondents who visited non-riding venues said they would ride an elephant in future. Eight respondents who rode an elephant said they would not ride an elephant again. Therefore, only ten participants (45.5%) who rode an elephant would choose to ride an elephant again.

Although 113 people stated whether they would ride an elephant in future, only 71 people provided a reason for why they would not participate in riding (Table 2). As this was an open-ended question, some respondents provided more than one reason, resulting in 86 reasons for why they would not participate in riding (21 themes when coded, see Supplementary Materials Table S4).

For respondents who stipulated a reason for not wanting to ride an elephant again or in the future, the most common reason was because they considered riding inhumane (*n* = 19, 22%) (“It is cruel and [elephants] should not be subjected to such treatments” and “Riding elephants is inhumane”). Respondents were also of the opinion that riding hurts the elephant or that it is “not right” (both *n* = 10, 11.6%) (“I don’t think it’s right to put them under that kind of strain”). Additionally, some respondents believed that elephants used for riding are treated badly or exploited (*n* = 9, 10.5%).

Eight respondents who had ridden an elephant on the day of the survey and said that they would not do it again provided multiple reasons for their decision (could report >1 reason). These included: the elephant does not like being ridden, they witnessed elephants being yelled at or physically manhandled (“Elephants didn’t like to ride and the trainer (did a) bad thing like crush(ed the elephant’s) skin with a little knife”), they did it once for the experience, and that riding was scary (all *n* = 2).

### 3.5. Satisfaction with the ETV

The majority of participants surveyed would re-visit the ETV they visited (*n* = 100, 77%), with only 23% stating they would not re-visit (*n* = 30). Two participants from non-riding venues did not respond. Based on trek type, 81.7% (*n* = 85) of respondents who visited a non-riding ETV would re-visit, 56.5% (*n* = 13) of participants who visited a bareback riding ETV would re-visit and two of the three participants who visited a seated riding venue would re-visit. All participants at two bareback riding venues and one non-riding venue would re-visit (see Supplementary Materials Table S5 for rankings). This was followed by three other high scoring non-riding venues where over 80% of respondents would re-visit. Conversely, at one bareback riding venue only 20% of respondents would re-visit, the lowest of the study.

Although 100 participants stated they would re-visit the ETV, only 88 provided a reason for why they would not take part in riding (Table 3). As this was an open-ended question, some respondents provided more than one reason, resulting in 146 reasons for why they would re-visit the ETV. Twenty-one themes were constructed based on respondents’ answers (see Supplementary Materials Table S6).

No respondents reported riding specifically as a reason to re-visit the same ETV, insofar that they enjoyed the activities or the experience in general. The most popular reason cited for wanting to return to the same ETV was because respondents felt the ETV cared for and treated the elephants humanely (*n* = 31, 21%) (“…elephants there are treated very well”), followed by respondents reporting they had fun and enjoyed themselves at the ETV (*n* = 23, 15.8%). Additional reasons stated for wanting to re-visit include the guides and ETV staff being friendly or “good” (*n* = 19, 13%) and a number of respondents regarded the visit as a once in a lifetime or good experience (*n* = 11, 7.5%).

Of the 30 participants who said they would not re-visit the same ETV, 29 respondents provided 37 reasons (15 themes when coded, see Supplementary Materials Table S7) (Table 4). Four respondents (10.8%) reported riding specifically as a reason not to re-visit the same ETV, stating that they wanted to make better choices and find an ETV (or ‘sanctuary’) that does not use chains or offer activities like riding and elephant tricks. The most commonly cited reasons for not wanting to return to the ETV was that they only intended to have this experience once, or that they would prefer to see elephants in their natural habitat (both *n* = 5, 13.5%) (“I think that elephants should be viewed from afar in their natural habitat”).

### 3.6. Satisfaction with the ETV’s Treatment of Elephants

Most respondents believe the ETV they visited treated the elephants appropriately (*n* = 102, 79.7%). Respondents were also unsure (*n* = 23, 18%) or did not believe the ETV they visited treated the elephants appropriately (*n* = 3, 2.3%). Based on trek type, the majority of respondents who visited a non-riding venue (*n* = 87, 85.3%), many respondents who visited a bareback riding venue (*n* = 14, 60.9%) and one respondent who visited a seated riding venue (33.3%) believed the ETV they visited cared for the elephants. Fourteen respondents from non-riding venues, eight from bareback riding venues and one from a seated riding venue were unsure. One respondent from all three venue types reported they did not believe the ETV they visited treated the elephants appropriately. At two bareback riding venues and two non-riding venues, all respondents believed the ETV they visited cared for the elephants. Respondents reported the lowest satisfaction (33%) at three ETVs. These venues represented all trek types, comprising of one non-riding venue, one bareback riding venue and one seated riding venue.

When the participants were asked to provide reasons for whether they believe the ETV treated the elephants appropriately, responses were provided by 81 participants who said “yes”, 21 who said “unsure” and 2 who said “no”. Of the 81 who believe the ETV cared for the elephants appropriately, 119 reasons were provided (Table 5; 29 themes when coded, see Supplementary Materials Table S8). Three respondents reported they believe the ETV treated the elephants appropriately because there is no riding at the venue or because the elephants are only ridden for a limited time. The most common reason provided by respondents was because the elephants were treated well and cared for (*n* = 28, 23.5%), followed by the knowledge and information provided by the guides (*n* = 10, 8.4%). Additional reasons include the respondents did not see anything that concerned them, or they did not witness any harm towards the elephants or see any wounds (both *n* = 9, 7.6%). Some respondents stated they believe the ETV treated its elephants appropriately but proceeded to give somewhat negative reviews before justifying the treatment of the elephants. For example, one respondent reasoned that the questionable treatment of the elephants is acceptable to enable tourist-elephant interactions: “It is obvious the care isn’t up to wildlife conservancy standards, but the treatment seemed justified in order for tourists to come”. While another acknowledged that there is room for improvement: ”I believe they could do better…when the elephants were tied up they did display some repetitive behaviour which was concerning”, but justified this treatment because there were “less commands, less chains, (and) less tricks”.

Of the 21 respondents who said they were unsure whether the ETV they visited treated the elephants appropriately, 32 reasons were provided (20 themes when coded, see Supplementary Materials Table S9). One respondent reported they were unsure whether the ETV treated the elephants appropriately because elephants are ridden at the venue. The most common reason provided by respondents was that they believe the elephants were chained for long periods (*n* = 5, 15.6%). This was followed by respondents thinking that the elephants appeared stressed (for example, “Moving the body left to right non-stop. They look anxious”) or believing there were too many tourists at the ETV (both *n* = 3, 9.4%).

Of the two respondents who do not believe the ETV they visited treated the elephants appropriately, they provided one reason each: “I think I saw a very small knife in (the) trainer’s hand”, and “Prefiero que esten en liberated” (Prefer elephants to be free).

## 4. Discussion

Tourists were surveyed about their preferences regarding the activities offered at ETVs and their satisfaction with their experience. In particular, the difference in responses between participants who visited elephant riding and non-riding venues was explored.

### 4.1. Reasons for Visiting ETVs

It is clear that participants visited different venues for similar reasons regardless of the type of activities they wished to participate in. Positive word-of-mouth endorsement from friends or reviews was the most popular reason for choosing the ETV (26.2%). This underlines the significance of international travel review forums, such as TripAdvisor, Lonely Planet and Rick Steves’ Travel Forum. While reviews can be helpful in determining which attraction to visit, most tourists have a flawed perception of the welfare implications of wildlife tourism venues; thus, many leave positive reviews for venues with lower welfare standards, further encouraging potential visitors to support these ETVs [38]. Alternatively, tourists also have the ability to post negative reviews for venues, informing potential visitors of the low welfare standards at the venue. In the absence of global regulatory authorities for wildlife tourism attractions, the income generated from tourists has become the deciding factor of what constitutes the acceptable use of animals in wildlife tourism [23]. Therefore, a negative review by visitors could impact the reputation of the ETV (as a venue’s reputation was the second most common reason for visiting an ETV), impact tourist revenue and encourage the venue’s management to improve their practices.

### 4.2. Participation in Elephant-Related Activities

It is important to understand tourist perspectives and preferences for elephant-related activities to discover where tourist education programs may be effective in contributing to improved welfare standards in elephant tourism [38]. Due to increasing public awareness, tourist preference for ETVs with higher welfare standards appears to be occurring. This preference is evident in this study as more respondents from non-riding and seated riding venues stated that they would prefer to participate in an elephant walk or to view elephants in the wild than any other activities. Respondents from bareback riding venues also expressed a preference to view elephants in the wild and ride bareback equally. Only one of the three respondents who participated in a seated ride listed this as an activity they wanted to experience. This low preference for seated riding may be due to the small sample of participants from these venues as these tourists appeared to be a family group who may have visited the ETV due to this one person’s desire. Educational programs could educate identified target groups on positive alternatives for popular activities, such as visiting non-riding instead of riding venues, and addressing misperceptions of the welfare of ETVs that many tourists hold.

### 4.3. Preferred and Least Preferred Activities

The respondents’ reasons for their preferred and least preferred activities indicate both ecocentric and anthropocentric worldviews were present among participants. Some respondents liked an activity because they believed the elephants enjoyed it or because it enabled them to get close to the elephant. Both reasons could inform ETVs’ targeting of tourists which could increase the number of close interactions between tourists and elephants.

Of those participants who rode an elephant, relatively fewer reported riding as their preferred or least preferred activity, which is likely due to fewer participants surveyed at riding venues compared to non-riding venues. Most respondents who liked riding did so because of a positive experience with elephants, such as the close contact it allowed with elephants or because it was a unique experience; although, one respondent stated they liked it for the feeling that riding an elephant gave them. Respondents who reported riding as their least preferred activity did so because of apparent negative experiences, including because the mahouts (elephant handlers) yelled at the elephants or they would have preferred to see the elephants in their natural habitat.

Interestingly, respondents reported bathing elephants in water as both their preferred and least preferred activities. This occurred because two separate questions were posed asking participants to specifically name their (1) preferred activity and (2) least preferred activity, instead of posing one question asking participants to name their preferred activity and deducing their least preferred activity from their responses. Feeding and walking with the elephants were also reported within both the top five preferred and least preferred activities. These activities were mainly liked for elephant-related reasons such as the ability for close contact and because the elephants appeared to enjoy the activity. Respondents disliked venues where they became wet or muddy, or because they were not prepared for a long walk, as well as venues where they believed elephants were forced to participate in an activity. One respondent questioned the purpose of elephant bathing, alluding to it as just another type of performance, as elephants would likely prefer to bathe themselves.

These results support other studies which found that tourist satisfaction was positively influenced by evidence that the elephants’ physical and mental welfare was supported, and thus emphasises the need for improved animal welfare in tourism venues [14,39]. Activities that fulfil tourists’ desire for close contact with elephants, such as bathing and feeding, are perceived as higher welfare alternatives to riding; however, they still require the elephants to be forcefully trained and controlled. Finding a balance between what is best for the welfare of each elephant and what is realistic in elephant tourism in Thailand could be a significant challenge.

### 4.4. Preferences for Elephant Riding

The common overarching theme that emerged for why respondents would not ride an elephant again or in future concerned the perceived poor treatment of elephants used for riding. This theme continued for those respondents who rode an elephant and said they would not do so again, as they reported more specific observations, such as elephants being yelled at or physically manhandled, and that the elephants did not appear to like the activity. This suggests that, for these participants who did not and would not ride an elephant, an attitude-behaviour gap does not exist as their actual behaviour aligns with their animal welfare attitudes and values. Conversely, as stated above, some respondents wanted to visit venues where elephants are treated well, but where they could also ride an elephant. This arguably contradictory view demonstrates a gap between their values and behaviour as they display knowledge of the mistreatment of elephants common in ETVs; however, their desire to experience elephant riding outweighed their desire to behave in harmony with their values [25].

Three of the four participants who visited bareback riding ETVs but chose not to ride were not aware that bareback riding occurred at the venue due to insufficient research or because one particular venue did not advertise bareback riding on their website. Thus, only one participant made an informed choice not to ride. This highlights the use of humane-washing schemes in marketing material common in elephant tourism. As there are no regulatory bodies for elephant tourism [23], it is also common for ETVs to use names like ’haven’, ’refuge’ and ’sanctuary’ to attract welfare-minded tourists [40]. However, due to the popularity and exploitation of these terms, they are often meaningless [40].

Less than half of the respondents who participated in bareback riding reported that they would ride an elephant again, and four respondents who visited non-riding venues on the day of the survey said that they would ride an elephant in future. These respondents did not provide reasons for why they would participate in elephant riding. Only one of the three participants who partook in a seated ride said they would ride an elephant again. Nevertheless, this demonstrates demand for elephant riding in Thailand and, as long as demand remains, bareback and seated riding venues will continue to operate and keep their elephants in low welfare standards.

Elephant tourism is a strong example supporting the argument that most people appear to accept the use of animals in tourism, provided they believe the animals are well cared for [41]. However, as is evident here, peoples’ attitudes differ, and disparities persist concerning the use of animals that people are willing to accept and reject [41].

### 4.5. Satisfaction with the ETV

Most respondents appeared satisfied with their visit, reporting that they would re-visit the same ETV. More respondents from the non-riding venues reported that they would re-visit than respondents from bareback and seated riding venues. This could be due to the participants who had negative experiences at a riding ETV and said they would not return. The two bareback riding venues and one non-riding venue where all respondents would revisit had lower numbers of participants which could have led to the high rate of respondents willing to re-visit. However, due to the varying nature of the trek types offered at these venues, these participants were after different experiences and possibly tailored their search for ETVs to reflect these desires, leading to high satisfaction rates.

Riding was not stated as a reason to re-visit but was specifically reported by some participants as a reason not to re-visit an ETV. The venue where the lowest number of respondents would re-visit was likely scored this way as it reflects the attitudes of the participants who visited with the expectation of walking with elephants and were previously unaware that it also offered bareback riding. It can also be argued that tourists who visit ETVs hoping for ’sanctuary’ conditions can be the most critical and report the lowest satisfaction rates when the experience does not match their expectations.

### 4.6. Satisfaction with the ETV’s Treatment of Elephants

The overall view that most respondents believed elephants were treated appropriately at the ETV they visited was likely influenced by the large number of participants from non-riding venues who held this opinion. Some respondents specifically stated that they believe the ETV treated the elephants appropriately because there was no riding or because the rides were short, and another stated they were unsure because elephants are ridden at the venue.

Two respondents displayed an anthropocentric worldview, stating that the treatment of the elephants was substandard but is justified to enable the close contact with elephants that tourists desire. This again demonstrates cognitive dissonance among visitors to ETVs as tourists disguise the moral implications of their decision and allow themselves to behave in a self-interested manner, possibly due to the activity’s strong appeal [25,42].

The three ETVs where only a fraction of respondents thought they treated the elephants well all varied in trek type. This suggests that the low ratings are due to the observed treatment of the elephants rather than in response to the activities offered. This is supported by the most common reasons cited by participants which concerned the prolonged chaining of elephants and that the elephants appeared stressed. Participants who were unsure whether the ETV treated the elephants appropriately were largely concerned with the presence and cause (chaining) of stereotypic behaviour, and the two respondents who did not believe the ETV treated its elephants well were concerned with the presence of abuse or because they would prefer the elephants to be free. However, the presence of stereotypic behaviour does not necessarily reflect the elephant’s current welfare conditions as the experiences that caused these behaviours may have occurred elsewhere. Tourists only received the information provided by the venue’s guides which, therefore, leaves those tourists concerned by the behaviour to make their own assumptions.

These results further substantiate claims that tourist satisfaction may be positively influenced by the elephant’s high welfare [14,39] and that most tourists do not intentionally ignore poor welfare if they are aware of it [43]. The variation in perceptions of elephant welfare between the three venue types also exists *within* each type of venue and, as most participants visited non-riding venues, it is most apparent in these participants’ responses as they were both for and against most aspects assessed, such as riding and close interactions. Therefore, much of the variation may be due to these participants. Further, the low number of participants willing to re-visit certain ETVs because of the observed poor treatment of elephants demonstrates tourist preference for venues with higher welfare standards.

## 5. Conclusions

This study investigated the role of tourist preference and satisfaction at riding and non-riding ETVs, contributing to an area requiring further research. We found that tourists visited different venues for similar reasons, primarily due to recommendations from friends and reviews, and because they perceived the venue to have a good reputation. These drivers for visitation were dominant regardless of the visitors’ preferred activities at the venues. The strong influence of international travel review forums over tourists’ choice of ETV demonstrates that negative reviews about elephant welfare could keep visitors away, and thus ultimately encourage venue management to implement higher welfare standards.

Tourist preference for higher welfare standards and satisfaction with their experience was demonstrated at ETVs where participants who observed poor treatment of elephants stated they would not return. Both anthropocentric and ecocentric worldviews were present among tourists, with a small number justifying the poor treatment of elephants as necessary to enable interactions between elephants and tourists. In future research, approaches such as focus groups or interviews could be utilised to better represent tourist preferences and satisfaction at seated riding venues and to better validate a small sample size. These methods could also be used to gain a more in-depth understanding of not just visitor perspectives but also that of other stakeholders, such as ETV operators, in elephant tourism in Thailand.

Both a questionnaire and participant observation were used in this research to minimise the effect of participant response bias. However, it remains possible that participants may have responded in a manner they believed the researcher desired. Increasing the mixed methods approach to data collection could further assist with this. The sample size was limited as the data were collected during a single month for logistical reasons. In addition, the study was undertaken during the tourist high season (November to February) and respondents came from a variety of locations. Future research should obtain a larger sample size, preferably including multiple sampling periods throughout the year. This would allow an examination of the relationships between the constructs employed here, such as tourist satisfaction and the intention to re-visit an ETV, through inferential statistical analysis. While the study occurred prior to the Covid-19 pandemic, the findings remain relevant as these activities are likely to resume when the pandemic is over.

Overall, participants’ chosen activities, ranking elephant walks and viewing elephants in the wild above elephant riding, reflected their preference for higher welfare standards. Tourists have the ability to influence the elephant tourism industry in Thailand by providing feedback in the form of reviews and recommendations, spreading awareness, and voting with their hip pocket by consciously visiting, and in turn financially supporting, ETVs with higher welfare standards.

## Figures and Tables

**Table 1:** Reasons given by respondents for choosing the ETV visited (*n* = 129).

Reasons (229)	Respondents (*n* = 129)	%
This park was recommended to me	60	26.2
I heard it has a good reputation	59	25.8
I researched elephant parks in the area and found that this best suited my needs	44	19.2
This park was advertised by a local travel agent	24	10.5
This park fit within my budget	16	7
Good welfare *	11	4.8
Not their first choice/their choice at all *	5	2.2
Involved with the ETV previously in some way *	4	1.7
This was the first place I saw	3	1.3
Wanted to ride elephants at an ETV that ‘cares’ for its elephants *	2	0.87
“To see if the elephants have a good conditional life” *	1	0.44

Number in parentheses in the first column (229) is the total reasons stated (participants could give >1 reason). * Denotes themes constructed from participants’ responses, all others were reasons included in the questionnaire.

**Table 2:** Top four reasons respondents gave for not riding an elephant in future.

Reasons (86)	Respondents (*n* = 71)	%
Riding is inhumane	19	22.09
Hurts the elephant	10	11.63
It’s “not right”	10	11.63
Riding elephants are treated badly or exploited	9	10.5

Number in parentheses in the first column is the total reasons stated (participants could give >1 reason).

**Table 3:** Top four reasons respondents gave for willingness to return to the ETV they visited.

Reasons (146)	Respondents (*n* = 88)	%
Felt the ETV cared for and treated the elephants humanely	31	21.23
Had fun and enjoyed themselves at the ETV	23	15.75
Guides and ETV staff were friendly or ‘good’	19	13.01
Once in a lifetime or good experience	11	7.5

Number in parentheses in the first column is the total reasons stated (participants could give >1 reason).

**Table 4:** Top three reasons respondents gave for not returning to the ETV they visited.

Reasons (37)	Respondents (*n* = 29)	%
Would only ever visit an ETV or have this experience once	5	13.5
Would prefer to see elephants in their natural habitat	5	13.5
Wanted to make better choices and find an ETV (or ’sanctuary’) that does not use chains or offer activities like riding and elephant tricks	4	10.8

Number in parentheses in the first column is the total reasons stated (participants could give >1 reason).

**Table 5:** Top four reasons respondents gave for believing the ETV they visited treats their elephants appropriately.

Reasons (119)	Respondents (*n* = 81)	%
Elephants were treated well and cared for	28	23.53
Knowledge and information provided by the guides	10	8.4
Did not see anything that concerned them	9	7.56
Did not witness any harm towards the elephants or did not see any wounds	9	7.56

Number in parentheses in the first column is the total reasons stated (participants could give >1 reason).

## Data Availability

The data presented in this study are available on request from the corresponding author. The data are not publicly available due to privacy restrictions.

## Supplementary Materials

The following are available online at https://www.mdpi.com/article/10.3390/ani11041094/s1. Table S1: Reasons respondents gave for choosing their preferred activity, Table S2: Reasons respondents gave for choosing their least preferred activity. Table S3: Respondent preferences, per venue type, for riding or not riding an elephant in future. Table S4: Reasons respondents gave for not riding an elephant in future. Table S5: Ranking of ETVs based on the tourists’ satisfaction with the venues. Table S6: Reasons respondents gave for willingness to return to the ETV they visited. Table S7: Reasons respondents gave for not returning to the ETV they visited. Table S8: Reasons respondents gave for believing the ETV they visited treats their elephants appropriately. Table S9: Reasons respondents gave for being unsure whether the ETV they visited treats their elephants appropriately, the Pre-Visit and Post-Visit Questionnaires, the Tourist Questionnaire Information Sheet, and the ETV Recruitment Email Script.

## References

[1] World Travel & Tourism Council (WTTC) Global Wildlife Tourism Generates Five Times More Revenue than Illegal Wildlife Trade Annually. https://www.wttc.org/about/media-centre/press-releases/press-releases/2019/global-wildlife-tourism-generates-five-times-more-revenue-than-illegal-wildlife-trade-annually/.

[2] Vanhaleweyk G. Tourism Statistics in Thailand 2000–2018. http://www.thaiwebsites.com/tourism.asp.

[3] World Travel & Tourism Council (WTTC) One in Five Thai Baht Spent is in Travel & Tourism, According to New WTTC Research. https://www.wttc.org/about/media-centre/press-releases/press-releases/2019/one-in-five-thai-baht-spent-is-in-travel-and-tourism-according-to-new-wttc-research/.

[4] Hughes P. (2001). Animals, values and tourism—structural shifts in UK dolphin tourism provision. Tour. Manag..

[5] Worwag S., Varga P., Zizka L. (2019). Tourists’ Ethical Concern for Dumbo- Elephant Tourism in Thailand. J. Travel Tour. Recreat..

[6] Daly N. (2019). Suffering Unseen: The Dark Truth Behind Wildlife Tourism. National Geographic.

[7] Carr N., Broom D.M. (2018). Tourism and Animal Welfare.

[8] Burns G.L., Green R., Lima I. (2017). Ethics and Responsibility in Wildlife Tourism: Lessons from compassionate conservation in the Anthropocene. Wildlife Tourism, Environmental Learning and Ethical Encounters.

[9] Newsome D., Dowling R., Moore S. (2004). Wildlife Tourism.

[10] Fennell D.A. (2013). Tourism and Animal Welfare. Tour. Recreat. Res..

[11] Kontogeorgopoulos N. (2009). Wildlife tourism in semi-captive settings: A case study of elephant camps in northern Thailand. Curr. Issues Tour..

[12] Schmidt-Burbach J., Ronfot D., Srisangiam R. (2015). Asian Elephant (*Elephas maximus*), Pig-Tailed Macaque (*Macaca nemestrina*) and Tiger (*Panthera tigris*) Populations at Tourism Venues in Thailand and Aspects of Their Welfare. PLoS ONE.

[13] Kontogeorgopoulos N. (2009). The Role of Tourism in Elephant Welfare in Northern Thailand. J. Tour..

[14] Cui Q., Xu H. (2019). Situating animal ethics in Thai elephant tourism. Asia Pac. Viewp..

[15] Polyapipat P., Loh A.L. (2015). Tourists’ understanding of the elephant business in the tourism industry- a study of international tourists in Chiang Mai Provence, Thailand. ABACODI J. Vision. Action. Outcome.

[16] Schmidt-Burbach J. (2016). Taken for a Ride: The Conditions for Elephants Used in Tourism in Asia.

[17] Oviedo-García M.A., Vega-Vázquez M., Castellanos-Verdugo M., Reyes-Guizar L.A. (2014). Tourist satisfaction and the souvenir shopping of domestic tourists: Extended weekends in Spain. Curr. Issues Tour..

[18] Mutanga C.N., Vengesayi S., Chikuta O., Muboko N., Gandiwa E. (2017). Travel motivation and tourist satisfaction with wildlife tourism experiences in Gonarezhou and Matusadona National Parks, Zimbabwe. J. Outdoor Recreat. Tour..

[19] Sun Y., Ma H., Chan E.H.W. (2018). A Model to Measure Tourist Preference toward Scenic Spots Based on Social Media Data: A Case of Dapeng in China. Sustainability.

[20] Kaffashi S., Yacob M.R., Clark M.S., Radam A., Mamat M.F. (2015). Exploring visitors’ willingness to pay to generate revenues for managing the National Elephant Conservation Center in Malaysia. For. Policy Econ..

[21] Castellanos-Verdugo M., Vega-Vázquez M., Oviedo-García M.A., Orgaz-Agüera F. (2016). The relevance of psychological factors in the ecotourist experience satisfaction through ecotourist site perceived value. J. Clean. Prod..

[22] Gursoy D., McCleary K.W., Lepsito L.R. (2007). Propensity to Complain: Effects of Personality and Behavioral Factors. J. Hosp. Tour. Res..

[23] Cohen S.A., Prayag G., Moital M. (2014). Consumer behaviour in tourism: Concepts influence, and opportunities. Curr. Issues Tour..

[24] Moorhouse T.P., D’Cruze N.C., Macdonald D.W. (2017). Unethical use of wildlife in tourism what’s the problem—Who is responsible and what can be done. J. Sustain. Tour..

[25] Bone J., Bone K., Markwell K. (2015). The Same Dart-Trick: The Exploitation of Animals and Women in Thailand Tourism. Animals and Tourism: Understanding Diverse Relationships.

[26] Moorhouse T.P., D’Cruze N.C., Macdonald D.W. (2017). The effect of priming, nationality and greenwashing on preferences for wildlife tourist attractions. Glob. Ecol. Conserv..

[27] Ballantyne R., Packer J., Falk J. (2011). Visitors’ learning for environmental sustainability testing short- and long-term impacts of wildlife tourism experiences using structural equation modelling. Tour. Manag..

[28] Worwag S.S., Varga P. Tourists’ animal welfare considerations—Elephant tourism in Thailand. Proceedings of the CAUTHE 2017: Time for Big Ideas? Re-Thinking the Field for Tomorrow.

[29] Bansiddhi P., Nganvongpanit K., Brown J.L., Punyapornwithaya V., Pongsopawijit P., Thitaram C. (2019). Management factors affecting physical health and welfare of tourist camp elephants in Thailand. PeerJ.

[30] Bansiddhi P., Brown J.L., Thitaram C. (2020). Welfare Assessment and Activities of Captive Elephants in Thailand. Animals.

[31] Norkaew T., Brown J.L., Bansiddhi P., Somgird C., Thitaram C., Punyapornwithaya V., Punturee K., Vongchan P., Somboon N., Khonmee J. (2019). Influence of season, tourist activities and camp management on body condition, testicular and adrenal steroids, lipid profiles, and metabolic status in captive Asian elephant bulls in Thailand. PLoS ONE.

[32] Norkaew T., Brown J.L., Thitaram C., Bansiddhi P., Somgird C., Punyapornwithaya V., Punturee K., Vongchan P., Somboon N., Khonmee J. (2019). Associations among tourist camp management, high and low tourist seasons, and welfare factors in female Asian elephants in Thailand. PLoS ONE.

[33] Taylor M. (2018). Elephant-Based Volunteer Tourism: An Exploration of Participant Experiences and Reflections on Captive Elephant Welfare in Thailand. Master’s Thesis.

[34] Thongma W., Guntoro B. (2011). Elephant camps and their impacts to community—Case study in Keud Chang, Chiang Mai Province, Thailand. Int. J. Agric. Travel Tour..

[35] Bansiddhi P., Brown J.L., Thitaram C., Punyapornwithaya V., Nganvongpanit K. (2020). Elephant Tourism in Thailand: A Review of Animal Welfare Practices and Needs. J. Appl. Anim. Welf. Sci..

[36] Rattan J.K., Eagles P.F.J., Mair H.L. (2012). Volunteer tourism: Its role in creating conservation awareness. J. Ecotourism.

[37] QSR International Pty NVivo Qualitative Data Analysis Software (Released in March 2018). https://www.qsrinternational.com/nvivo-qualitative-data-analysis-software/home.

[38] Braun V., Clarke V. (2006). Using thematic analysis in psychology. Qual. Res. Psychol..

[39] Moorhouse T.P., Dahlsjo C.A., Baker S.E., D’Cruze N.C., Macdonald D.W. (2015). The Customer Isn’t Always Right-Conservation and Animal Welfare Implications of the Increasing Demand for Wildlife Tourism. PLoS ONE.

[40] Lee H.-S. (2015). Measurement of visitors’ satisfaction with public zoos in Korea using importance-performance analysis. Tour. Manag..

[41] Winders D.J. (2017). Captive Wildlife at a Crossroads—Sanctuaries, Accreditation, and Humane-Washing. Anim. Stud. J..

[42] Fennell D.A. (2012). Tourism, animals and utilitarianism. Tour. Recreat. Res..

[43] Tenbrunsel A.E., Messick D.M. (2004). Ethical fading: The role of self-deception in unethical behaviour. Soc. Justice Res..

