# Supplementary material for: How Tourist Preference and Satisfaction Can Contribute to Improved Welfare Standards at Elephant Tourism Venues in Thailand
Source: Animals (Basel). 2021 Apr 12;11(4):1094. doi: 10.3390/ani11041094 (PMC8069915; doi:10.3390/ani11041094)
Supplement: Supplementary file 1 [file animals-11-01094-s001.pdf]

animals

MDPI

*Supplementary Material*

# How Tourist Preference and Satisfaction Can Contribute to Improved Welfare Standards at Elephant Tourism Venues in Thailand

**Emily K. Flower *, Georgette Leah Burns and Darryl N. Jones**

Environmental Futures Research Institute and School of Environment and Science, Griffith University, Nathan, Australia ; leah.burns@griffith.edu.au (G.L.B.); d.jones@griffith.edu.au (D.N.J.)
* Correspondence: emily.flower@griffithuni.edu.au

**Citation:** Flower, E.K.; Burns, G.L.; Jones, D.N. How Tourist Preference and Satisfaction Can Contribute to Improved Welfare Standards at Elephant Tourism Venues in Thailand. *Animals* **2021**, *11*, 1094. https://doi.org/10.3390/ani11041094

Academic Editor: Pia Lucidi

Received: 10 March 2021
Accepted: 6 April 2021
Published: 12 April 2021

**Publisher's Note:** MDPI stays neutral with regard to jurisdictional claims in published maps and institutional affiliations.

**Copyright:** © 2021 by the authors. Submitted for possible open access publication under the terms and conditions of the Creative Commons Attribution (CC BY) license (http://creativecommons.org/licenses/by/4.0/).

## Tables

**Table S1.** Reasons respondents gave for choosing their preferred activity. Additional details for Table 5. in the text. When 'reason frequency' is nil, no reason was provided by respondents.

| Preferred Activity (n = 75) | Reason Frequency |
|---|---|
| **Activities (Intentionally Observed)** | |
| Bathing in mud | 6x Close contact with elephants |
| | 4x Elephants appeared happy or to like the activity |
| | 3x 'Connected' with elephants |
| | 2x The activity was fun |
| | 2x Able to interact with the elephants |
| | 1x Elephants in natural environment |
| | 1x Beautiful scenery |
| | 1x Elephants 'didn't seem to mind' |
| Bathing in river or waterfall | 10x Elephants appeared happy or to like the activity |
| | 9x Close contact with elephants |
| | 7x The activity was fun |
| | 4x Able to interact with the elephants |
| | 3x Felt like they were 'helping' or caring for the elephants |
| | 3x It was a special or unique experience |
| | 2x Elephants in natural environment |
| | 2x Beautiful scenery |
| | 2x 'Connected' with elephants |
| | 1x Participant felt safe |
| Feeding | 9x Close contact with elephants |
| | 6x Elephants appeared happy or to like the activity |
| | 3x Elephants were behaving naturally or weren't being commanded |
| | 3x Felt like they were 'helping' or caring for the elephants |
| | 2x To observe how elephants eat and behave |
| | 2x The activity was fun |
| | 2x Able to interact with the elephants |
| Riding | 3x It was a special or unique experience |
| | 1x Elephants 'didn't seem to mind' |
| | 1x Made the participant feel important e.g. 'like a King' |
| | 1x The activity was fun |
| | 1x Close contact with elephants |
| Taking photos | NIL |
| Walking with the elephants | 2x Elephants were behaving naturally or weren't being commanded |
| | 1x To observe how elephants eat and behave |
| | 1x 'Connected' with elephants |
| | 1x It was a special or unique experience |
| | 1x Spiritual experience |
| | 1x Close contact with elephants |
| **Interactions** | |
| Interacting with or touching elephants | 1x Elephants appeared happy or to like the activity |
| | 1x Able to interact with the elephants |
| | 1x Close contact with elephants |
| 'Organic' interactions over organised ones | 1x Elephants were behaving naturally or weren't being commanded |
| Spend time with elephants | NIL |
| **Observing elephants** | |
| Observing elephants - general | 2x Elephants appeared happy or to like the activity |
| | 1x Elephants in natural environment |
| Observing elephants bathe in mud | NIL |
| Observing elephants bathe in water | 2x Elephants appeared happy or to like the activity |
| | 1x Elephants were behaving naturally or weren't being commanded |
| Observing elephants eat | 1x Elephants appeared happy or to like the activity |

| | |
|---|---|
| | 1x Able to interact with the elephants |
| Observing elephants in their natural habitat | NIL |
| Observing elephants play (together or alone) | 2x Elephants appeared happy or to like the activity<br>1x Elephants were behaving naturally or weren't being commanded |
| Observing elephants throw dirt on themselves | 1x The activity was fun |
| **Other** | |
| Baby elephant or nursery | 1x It was a special or first-time experience<br>1x The elephants are cute |
| 'Everything' - no other description (at bareback venue) | NIL |
| 'Everything' - no other description (at non-riding venue) | NIL |
| Making vitamin balls for the elephants | 1x Felt like they were 'helping' or caring for the elephants |
| Non-elephant activities | NIL |
| **Total** | **123 reasons** |

**Table S2.** Reasons respondents gave for choosing their least preferred activity. Additional details Figure 7. in the text. When the 'reason frequency' is nil, no reason was provided by respondents.

| Least Preferred Activity (n = 40) | Reason Frequency |
|---|---|
| **Didn't have a least preferred - liked them all** | |
| Participants who visited bareback riding venues | NIL |
| Participants who visited non-riding venues | NIL |
| Participants who visited seated riding venues | NIL |
| **Activities (intentionally observed)** | |
| Bathing in mud | 2x Felt the elephants were forced to participate in the activity<br>2x They got wet or muddy<br>1x The activity felt like a performance<br>1x Would have preferred to watch the elephants bathe themselves<br>1x Felt weird around the guides<br>1x Personal health - got a rash or fear of sickness |
| Bathing in river or waterfall | 5x Felt the elephants were forced to participate in the activity<br>3x They got wet or muddy<br>2x Felt the elephants didn't enjoy the activity<br>1x The activity felt like a performance<br>1x Would have preferred to watch the elephants bathe themselves<br>1x Elephant not doing what the participant wanted<br>1x Personal health - got a rash or fear of sickness<br>1x Activity did not involve elephants |
| Close contact activities | NIL |
| Elephant tricks | NIL |
| Elephant walk | 4x Unprepared for a hike or unfit<br>1x Felt the elephants were forced to participate in the activity<br>1x Felt the elephants didn't enjoy the activity<br>1x Felt the elephants were not treated well during the activity<br>1x Hot weather |
| Feeding | 2x Chained in the sun or chained in general<br>2x Felt they did not interact enough with the elephants<br>1x The activity felt like a performance<br>1x Didn't find the activity interesting |
| Riding - bareback | 2x Felt the activity hurts the elephants or is 'inhumane and wrong'<br>1x Given too many commands or being yelled at by the mahouts<br>1x Elephants not in their natural habitat |
| Riding - seated | 1x The activity felt dangerous |
| Taking posed photos with the elephants | NIL |

| | |
|---|---|
| | **Guides & mahouts** |
| Commands from mahouts | NIL |
| Information from the guide | 1x Didn't understand the guide's speech (non-native English speaker)<br>1x Wanted more educational information from the guide |
| | **Other** |
| Information from the guide | NIL |
| Making vitamin balls for the elephants | 1x Unsure of the purpose of the activity |
| Observing elephants being treated for captivity-related injuries | NIL |
| Participant expected to give more elephants a mud bath | NIL |
| Seeing the elephants chained | NIL |
| Throwing mud at the participants | NIL |
| Non-elephant activities | 2x Long journey to the ETV |
| | 1x Personal health - got a rash or fear of sickness<br>1x Would have liked to spend more time walking instead of long lunch |
| **Total** | **49 reasons** |

**Table S3.** Respondent preferences, per venue type, for riding or not riding an elephant in future.

| | **Venue Type Would Visit in Future (n = 132)** | | | | |
|---|---|---|---|---|---|
| **Venue Type Visited** | **Non-Riding Venue** | **Bareback Riding Venue** | **Seated Riding Venue** | **Riding Venue (Unspecified)** | **Non-Response** |
| Non-riding venue | 84 | | | 4 | 18 |
| Bareback riding venue | 6 | 5 | | 8 | |
| Bareback riding venue (but did not ride) | 2 | 4 | | | 1 |
| Seated riding venue | 2 | | | 1 | |
| **Total** | **94** | **6** | **0** | **13** | **19** |

**Table S4.** Reasons respondents gave for not riding an elephant in future. Additional details for Table 8 in the text.

| Reasons Provided for not Wanting to Ride an Elephant again or in Future (n = 71) | Reason Frequency | Percent |
|---|---|---|
| **Elephants unable to be natural** | | |
| Desire to experience elephants as naturally as possible | 5 | 5.8 |
| It is not normal | 2 | 2.3 |
| Should just observe them or we should not ride animals | 2 | 2.3 |
| **For the elephant** | | |
| Hurts the elephant | 10 | 11.6 |
| Do not want to make elephant mad | 1 | 1.16 |
| Felt there were issues with the elephants' food | 1 | 1.16 |
| It's 'not right' | 10 | 11.6 |
| Riding elephants are treated badly or exploited | 9 | 10.5 |
| Riding is dangerous for the elephant | 1 | 1.16 |
| Riding is inhumane | 19 | 22.09 |
| The elephant 'doesn't like it' | 5 | 5.8 |
| Witnessed elephants being yelled at or physically manhandled | 2 | 2.3 |
| Won't ride an elephant because of the training process or because they 'have been abused' | 5 | 5.8 |
| **For the participant** | | |
| Desire to protect elephants | 1 | 1.16 |
| Did it for the experience | 2 | 2.3 |
| Do not want to encourage lots of other tourists to do it | 1 | 1.16 |
| 'I don't like it' or heard bad stories | 3 | 3.5 |
| It's a tourist activity – do not really meet the elephant | 1 | 1.16 |
| Riding is unnecessary or have no desire for it | 3 | 3.5 |
| Riding was scary | 2 | 2.3 |
| Will never ride an elephant and wants to spread the message | 1 | 1.16 |
| **Total** | **86** | |

**Table S5.** Ranking of ETVs based on the tourists' satisfaction with the venues. '*' denotes the venues that were visited once, and '^' denotes the venue at which two participants did not respond to this question.

| Rank | ETV Type | Tourist Satisfaction Percent | No. of Participants (n = 130) |
|---|---|---|---|
| 1 | Bareback riding | 100 | 5 |
| 1 | Bareback riding | 100 | 2 |
| 1 | Non-riding | 100 | 7* |
| 4 | Non-riding | 89.5 | 19 |
| 5 | Non-riding | 86.67 | 31^ |
| 6 | Non-riding | 80.56 | 36* |
| 7 | Bareback riding | 66.67 | 6 |
| 7 | Seated riding | 66.67 | 3* |
| 9 | Non-riding | 62.5 | 8 |
| 10 | Bareback riding | 33.3 | 4* |
| 10 | Non-riding | 33.3 | 3* |
| 12 | Bareback riding | 20 | 6 |

**Table S6.** Reasons respondents gave for willingness to return to the ETV they visited. Additional details for Table 9 in the text.

| Reasons for Re-Visiting an ETV (n = 88) | Reason Frequency | Percent |
|---|---|---|
| Diverse activities or felt the elephants liked the activities | 2 | 1.37 |
| Educational | 9 | 6.16 |
| Liked the ETV | 8 | 5.48 |
| Repeat visitor | 1 | 0.68 |
| **Guides or staff** | | |
| Guides and ETV staff were friendly or 'good' | 19 | 13.01 |
| Guides were knowledgeable or answered their questions re: treatment of elephants | 8 | 5.48 |
| Staff 'love' the elephants or believed they had a good relationship with the elephants | 3 | 1.37 |
| **For the elephant** | | |
| Elephants seemed happy or allowed to be free at night | 6 | 4.11 |
| Felt like spent the day with elephants in their natural habitat | 8 | 5.48 |
| Felt the ETV cared for and treated the elephants humanely | 31 | 21.23 |
| The visit supports the elephants | 1 | 0.68 |
| **For the participant** | | |
| Close contact or interactions with elephants | 7 | 4.79 |
| ETV is therapeutic | 1 | 0.68 |
| Felt a part of the wildlife | 1 | 0.68 |
| Felt safe | 1 | 0.68 |
| Liked the elephants | 6 | 4.11 |
| **Good experience** | | |
| Had fun and enjoyed themselves at the ETV | 23 | 15.75 |
| Once in a lifetime or good experience | 11 | 1.5 |
| **Total** | **146** | |

**Table S7.** Reasons respondents gave for not returning to the ETV they visited. Additional details for Table 10 in the text.

| Reasons for not Re-Visiting an ETV (n = 29) | Reason Frequency | Percent |
|---|---|---|
| **For the elephant** | | |
| Do not believe the elephants are treated well or have a 'good life' | 3 | 8.1 |
| Elephants were chained or sad to see them chained | 3 | 8.1 |
| Felt like the elephants were forced to do things they didn't want to do | 2 | 5.4 |
| Witnessed elephants being yelled at or physically manhandled | 1 | 2.7 |
| **For the participant** | | |
| Do not think 'sanctuaries' in Thailand care for their elephants or do not believe the guides are telling the whole truth | 2 | 5.4 |
| Learnt new information and now believe elephants should be in a sanctuary | 1 | 2.7 |
| Scared of elephants | 1 | 2.7 |
| Too far to travel or won't be back to Thailand | 3 | 8.1 |
| Too many visitors or wanted a more personal experience or to volunteer for a longer time | 2 | 5.4 |
| Wanted to make better choices and find an ETV (or 'sanctuary') that does not use chains or offer activities like riding and elephant tricks | 4 | 10.8 |
| Want to visit another ETV or prefer another ETV | 2 | 5.4 |
| Wasn't what participant expected | 2 | 5.4 |
| Would like to help elephants in a different way | 1 | 2.7 |
| Would only ever visit an ETV or have this experience once | 5 | 13.5 |
| Would prefer to see elephants in their natural habitat | 5 | 13.5 |
| **Total** | **37** | |

**Table S8.** Reasons respondents gave for believing the ETV they visited treats their elephants appropriately. Additional details for Table 11 in the text.

| ETV does treat Elephants appropriately (n = 81) | Reason Frequency | Percent |
|---|---|---|
| Believe elephants are happy or relaxed or look healthy | 8 | 6.7 |
| Believe elephants are part of the mahout's or venue's family or are dedicated staff | 6 | 5.04 |
| Did not see anything that concerned them | 9 | 7.56 |
| Did not witness any harm towards the elephants or did not see any wounds | 9 | 7.56 |
| Elephants are able to be elephants or can do what they like | 4 | 3.36 |
| Elephants were treated well and cared for | 28 | 23.53 |
| Elephants had free time after finished the activities or could roam free on off days | 2 | 1.68 |
| Elephants have specialised individual health care or ETV makes medicine for them | 3 | 2.52 |
| Elephants live in a more natural setting compared to other ETVs or have freedom | 8 | 6.7 |
| Elephants received a lot of attention | 1 | 0.84 |
| Elephants were well fed or given extra water by hose | 6 | |
| Knowledge and information provided by the guides | 10 | 8.4 |
| It looks like a 'real' sanctuary... | 1 | 0.84 |
| 'It's a feeling' | 1 | 0.84 |
| Kept calves with their mothers or calves could interact with other elephants | 2 | 1.68 |
| Large property or enclosure | 1 | 0.84 |
| Mahouts do not yell at elephants or carry sticks | 1 | 0.84 |
| No chaining | 2 | 1.68 |
| No elephant show | 1 | 0.84 |
| No hook or punishment | 2 | 1.68 |
| No riding or elephants only ridden for a limited time | 3 | 2.52 |
| Not sanctuary level care, but treatment seemed justified to allow for tourists to visit | 3 | 2.52 |
| **Issues that concerned participants, but they still believe the ETV cares for its elephants** | | |
| Didn't like that mahouts pulled on the elephants' ears and want them to let the elephants do what they want | 1 | 0.84 |
| Elephants made to learn tricks | 1 | 0.84 |
| Elephants were chained or for long periods | 2 | 1.68 |
| Observed stereotypical behaviour | 1 | 0.84 |
| Sceptical re: where elephants are before and after activities – do not believe they roam free | 1 | 0.84 |
| Too many tourists - elephants seemed stressed | 1 | 0.84 |
| Wish the elephants could be in the wild | 1 | 0.84 |
| TOTAL | 119 | |

**Table S9.** Reasons respondents gave for being unsure whether the ETV they visited treats their elephants appropriately.

| Unsure whether ETV Treats Elephants Appropriately (n = 21) | Reason Frequency | Percent |
|---|---|---|
| Believe elephants are treated appropriately 'for what it is' – ETV seemed to care for the elephants | 1 | 3.13 |
| Believe the ETV profits from the elephants or recently 'purchased' elephants | 2 | 6.25 |
| Calves were separated from their mothers | 1 | 3.13 |
| Didn't like the elephants' enclosure | 2 | 6.25 |
| 'Don't know why, just don't feel good' | 1 | 3.13 |
| Elephant still used for rides has killed people | 1 | 3.13 |
| Elephants treated well but felt the elephants were forced to do the activities or elephants are still a show | 2 | 6.25 |
| Elephants were chained for long periods | 5 | 15.62 |
| Elephants were ridden | 1 | 3.13 |
| Mahout had a small knife | 1 | 3.13 |

| | | |
|---|---|---|
| Not fed enough | 1 | 3.13 |
| Elephants appeared stressed | 3 | 9.38 |
| Too many tourists | 3 | 9.38 |
| Unsure if the elephants should just be in the wild | 1 | 3.13 |
| Unsure what 'appropriate' treatment is - did not witness elephants being harmed | 1 | 3.13 |
| Witnessed perceived harm or cruelty | | |
| Did not like how some of the mahouts behaved towards the elephants - wanted an explanation | 2 | 6.25 |
| Mahouts poked the elephants with bamboo | 1 | 3.13 |
| Mahouts pulled the elephants' ears | 1 | 3.13 |
| Mahouts yelled at elephants | 1 | 3.13 |
| Observed marks on elephants that seem recent | 1 | 3.13 |
| **Total** | 32 | |

**Questionnaires** (format condensed here)

Pre-Visit Questionnaire

***Please Fill in Your Initials & Year of Birth Here*** → Questionnaire#: ____________________

1. In what region is your permanent residence?

☐ East Asia ☐ The Americas ☐ Oceania ☐ Africa
☐ South Asia ☐ Europe ☐ Middle East

2. How do you identify?

☐ Male ☐ Female ☐ Prefer not to say ☐ Other________________

3. What is your age?

☐ 18-29 years old ☐ 40-49 years old ☐ 60-69 years old
☐ 30-39 years old ☐ 50-59 years old ☐ 70 and over

4. What is the highest level of education that you have attained?

☐ Less than high (secondary) school ☐ High (secondary) school
☐ College Diploma/ Tafe Certificate ☐ Undergraduate Degree
☐ Masters/Honours Degree ☐ Doctorate

5. I currently own a pet or have owned a pet:

☐ Yes ☐ No

6. How long is your vacation in Thailand?

☐ Less than 1 week ☐ 2 – 3 weeks ☐ More than 4 weeks
☐ 1 – 2 weeks ☐ 3 – 4 weeks

7. Please check all the activities that you have participated in, or wish to participate in, during your visit to Thailand:

☐ Cultural Attractions e.g. temples, hill-tribes, hiking, theatre etc. ☐ Wildlife Viewing
☐ Elephant Trekking ☐ Wildlife Reserve/Sanctuary ☐ Shopping
☐ Water Activities e.g. cruise, snorkelling, beaches etc. ☐ Medical Procedures
☐ Tiger Temples ☐ Sport e.g. Thai boxing ☐ Cooking Classes
☐ Botanical Gardens ☐ Other (Please Specify) ______________________________

**8.** What is your primary reason for visiting Thailand?

__________________________________________________________________________

__________________________________________________________________________

__________________________________________

**9.** Please check all reasons for visiting this particular elephant park:

☐ I heard it has a good reputation ☐ I researched elephant parks in the area and found that this best suited my needs
☐ This park was recommended to me ☐ This park fit within my budget
☐ This was the first place I saw ☐ This park was advertised by a local travel agent
☐ Other (Please Specify) ____________________________________________________

**10.** Is this your first visit to an elephant park?

☐ Yes ☐ No

**11.** If this is not your first visit, how many times have you been to an elephant park?

☐ Two ☐ Three ☐ Four ☐ More than 4 times

**12.** How long do you expect to stay at the elephant park?

☐ Day Visit ☐ Two days (1 overnight) ☐ Three days (2 overnight)
☐ Other (Please Specify) ____________________________________________________

**13.** Please check all the elephant-related activities that you have participated in, or would like to participate in, during your visit to Thailand:

☐ Elephant Walks (no riding) ☐ Elephant Shows e.g. painting, soccer, orchestra

☐ Elephant Ride Bareback ☐ Visit a Zoo

☐ Elephant Ride with Howdah (seat) ☐ Feed Street Elephants

☐ Visit a National Park/Sanctuary to View Elephants in the Wild

☐ Other (Please Specify) ________________________________

☐ I would not participate in any elephant activities listed above

***Please circle the number that best corresponds with your response to the statements below:***

| **Question** | | **Strongly Disagree** | | | | **Strongly Agree** |
|---|---|---|---|---|---|---|
| **14** | I enjoy viewing wildlife in a natural setting. | 1 | 2 | 3 | 4 | 5 |
| **15** | I am visiting the park because I would like to have close interactions with elephants. | 1 | 2 | 3 | 4 | 5 |
| **16** | I would like to learn more about the Asian elephant. | 1 | 2 | 3 | 4 | 5 |
| **17** | I enjoy watching elephants perform tricks. | 1 | 2 | 3 | 4 | 5 |
| **18** | I would like to volunteer with organisations back home that advocate and protect animal rights (i.e. a shelter). | 1 | 2 | 3 | 4 | 5 |
| **19** | I would like to donate money to animal conservation organisations. | 1 | 2 | 3 | 4 | 5 |
| **20** | The welfare and protection of animals is important to me. | 1 | 2 | 3 | 4 | 5 |
| **21** | I am aware of the conservation issues surrounding elephants in Thailand. | 1 | 2 | 3 | 4 | 5 |
| **22** | I am aware of the laws, regulations, and policies that govern elephant rights in Thailand. | 1 | 2 | 3 | 4 | 5 |
| **23** | I would <u>not</u> change the current policies governing elephants in Thailand. | 1 | 2 | 3 | 4 | 5 |
| **24** | I feel that elephants are treated humanely in Thailand. | 1 | 2 | 3 | 4 | 5 |
| **25** | I would like to volunteer at an elephant park. | 1 | 2 | 3 | 4 | 5 |
| | **Identify <u>how important</u> these factors are for elephant conservation:** | | | | | |
| **Question** | | **Not Important** | | | | **Extremely Important** |
| **26** | Laws/Protective Legislation | 1 | 2 | 3 | 4 | 5 |
| **27** | Fundraising | 1 | 2 | 3 | 4 | 5 |
| **28** | Creating Public Awareness | 1 | 2 | 3 | 4 | 5 |
| **29** | Government Support | 1 | 2 | 3 | 4 | 5 |
| **30** | Non-Governmental Organisation (NGO) | 1 | 2 | 3 | 4 | 5 |
| **31** | Community Support | 1 | 2 | 3 | 4 | 5 |
| **32** | Volunteers | 1 | 2 | 3 | 4 | 5 |

Post-Visit Questionnaire

***Please Fill in Your Initials & Year of Birth Here*** → Questionnaire #:___________________

1. How long did you stay at the elephant park?

☐ Day Visit ☐ Three Days (2 Overnight)

☐ Two days (1 Overnight) ☐ I decided to stay on as a volunteer

2. What was your favourite activity that the elephant park offered today? Why?

______________________________________________________________________

______________________________

3. What was your least favourite activity that the elephant park offered today? Why?

______________________________________________________________________

______________________________

4. Did you ride an elephant today? (Please check all that apply)

☐ Yes ☐ No

☐ With Howdah (seat) ☐ Bareback

5. Would you ride an elephant again?

☐ Yes ☐ No

☐ With Howdah (seat) ☐ Bareback

6. If no, please explain

______________________________________________________________________

____________________________________________________________

7. Would you visit this park again?

☐ Yes ☐ No

8. Why/why not?

______________________________________________________________________

______________________________

**9.** From what you have seen here today, are you of the opinion that this park cares for their elephants appropriately?

☐ Yes ☐ No ☐ Unsure

**10.** Why/why not? (did you see anything today that concerned you?).

______________________________________________________________________________

__________________________________________

***Think about the experiences you just had at the elephant park and circle the number that best corresponds with your response to the statements below:***

| **Question** | | **Strongly Disagree** | | | | **Strongly Agree** |
|---|---|---|---|---|---|---|
| **11** | I enjoy viewing wildlife in a natural setting. | 1 | 2 | 3 | 4 | 5 |
| **12** | I am visiting the park because I would like to have close interactions with elephants. | 1 | 2 | 3 | 4 | 5 |
| **13** | I would like to learn more about the Asian elephant. | 1 | 2 | 3 | 4 | 5 |
| **14** | I enjoy watching elephants perform tricks. | 1 | 2 | 3 | 4 | 5 |
| **15** | I would like to volunteer with organisations back home that advocate and protect animal rights (i.e. a shelter). | 1 | 2 | 3 | 4 | 5 |
| **16** | I would like to donate money to animal conservation organisations. | 1 | 2 | 3 | 4 | 5 |
| **17** | The welfare and protection of animals is important to me. | 1 | 2 | 3 | 4 | 5 |
| **18** | I am aware of the conservation issues surrounding elephants in Thailand. | 1 | 2 | 3 | 4 | 5 |
| **19** | I am aware of the laws, regulations, and policies that govern elephant rights in Thailand. | 1 | 2 | 3 | 4 | 5 |
| **20** | I would <u>not</u> change the current policies governing elephants in Thailand. | 1 | 2 | 3 | 4 | 5 |
| **21** | I feel that elephants are treated humanely in Thailand. | 1 | 2 | 3 | 4 | 5 |
| **22** | I would like to volunteer at an elephant park. | 1 | 2 | 3 | 4 | 5 |
| | **Identify how important these factors are for elephant conservation:** | | | | | |
| **Question** | | **Not Important** | | | | **Extremely Important** |
| **23** | Laws/Protective Legislation | 1 | 2 | 3 | 4 | 5 |
| **24** | Fundraising | 1 | 2 | 3 | 4 | 5 |
| **25** | Creating Public Awareness | 1 | 2 | 3 | 4 | 5 |
| **26** | Government Support | 1 | 2 | 3 | 4 | 5 |
| **27** | Non-Governmental Organisation (NGO) | 1 | 2 | 3 | 4 | 5 |
| **28** | Community Support | 1 | 2 | 3 | 4 | 5 |
| **29** | Volunteers | 1 | 2 | 3 | 4 | 5 |

**Tourist Questionnaire Information Sheet**

**The relationships between Asian elephants and people at elephant parks in Thailand.**

**HARD COPY QUESTIONNAIRE COVERSHEET**

| | |
|---|---|
| **Who is conducting the research** | Chief Investigators: (Names)<br>Student Researcher: (Name)<br><br>University Name, Country<br>Phone: (International number)<br>Email: (Chief Investigator email) |

**Why is the research being conducted?**

This research investigates elephant welfare in Chiang Mai, Thailand. It aims to identify the pre- and post-trip perceptions and opinions of international tourists and volunteers experiencing encounters with elephants in Thailand, and to determine how ecotourism affects the welfare of captive Thai elephants. This research forms a component of the student's academic program.

**What you will be asked to do**

As a participant in this research, you are asked to agree to participate in a questionnaire about your experiences with elephants and elephant parks in Thailand, in particular your attitudes towards, and perceptions of, elephant health and wellbeing. The questionnaire will take about 10 minutes.

**The expected benefits of the research**

This research is expected to contribute to scientific knowledge about the improvement of elephant welfare in Thailand, and ways to increase tourist satisfaction. In addition, we hope to educate the general public on the implications of training elephants (and in turn, other wild animals) to perform unnatural behaviours for the entertainment of tourists.

**Risks to you**

There are no foreseeable risks to you. Should you have any doubts about risks, you are able to discontinue the questionnaire at any point in time by voicing your concern to the researcher.

**Your confidentiality**

All data collected during this study will be de-identified. This means that data will be presented in research publications in a manner that will not identify you or allow you to be identified by third parties.

As required by (University name), all research data (questionnaire responses and analysis) will be retained in a password protected electronic file at (University name) for a period of five years, before being destroyed.

**Your participation is voluntary**

Your participation in this questionnaire is entirely voluntary. You are free to withdraw from the study, and can discontinue answering the questions posed to you at any time.

**Mechanism for distribution and return**

Questionnaires are distributed to people visiting the elephant parks, generally by bus. You may return your completed questionnaire to the person who distributed it.

The results of this study will only be used in scientific literature. No feedback will be given to participants outside of this.

**Questions / further information**

Should you have any questions or require further information, you are welcome to contact (Chief Investigator name and email), (Chief Investigator name email), or (Student Researcher name and email).

**The ethical conduct of this research**

(University name) has cleared this project regarding any ethical concerns (ref no.). (University name) conducts research in accordance with the *National Statement on Ethical Conduct in Human Research*. If you have any concerns or complaints about the ethical conduct of the research project, you may contact the Manager, Research Ethics on (phone number) or **(email address)**.

**Privacy Statement**

The conduct of this research involves the collection, access and/or use of your identified personal information. The information collected is confidential and will not be disclosed to third parties without your consent, except to meet government, legal or other regulatory authority requirements. A de-identified copy of this data may be used for other research purposes including publishing openly (e.g., in an open access repository). However, your anonymity will at all times be safeguarded. For further information consult the University's Privacy Plan at (website) or telephone (number).

**ETV Recruitment Email Script**

Dear Sir/Madam,

My name is (Researcher name), and I am a PhD candidate at (University name), located in (State, Country).

My study investigates the attitudes of international volunteers/tourists travelling to Thailand to interact with elephants in the ecotourism industry. This study will involve personal visits to a number of elephant camps/parks in the Chiang Mai area to describe the range of interactions between visitors and elephants and to assess the experience of tourists with the park and the activities on offer. I plan to visit between Nov 2017- April 2018, however, I would only need to visit (Elephant tourism venue [ETV] name) for two days during this time. A number of parks have already agreed to participate in this study, and it would be great if you joined as well!

I do not require any special requirements as I hope to simply observe the interactions between the elephants and the guests of the park throughout the day. I am hoping to participate in activities like any other guest of the park, and as such would of course pay the entry fee associated with partaking in the day's activities.

The purpose of this email is to seek the permission of the staff at (ETV name) to undertake this research at the park. Please find attached my permission letter from the NRCT to perform this research in Thailand. I wish to visit your park as part of this study; however, I am aware that visitor placement may have been filled well into next year. This does not pose a problem as I would not require accommodation on your premises, simply to join in the daily activities.

Please do not hesitate to contact me if you have any questions, and I look forward to hearing from you soon.

Kind Regards,

(Researcher name)
